# Poor Quality of Sleep among Healthcare Workers in a Tertiary Care Centre

**DOI:** 10.31729/jnma.8435

**Published:** 2024-02-29

**Authors:** Bikram Kafle, Suraj Tiwari, Arpana Pokhrel, Rajesh Shrestha, Yashoda Bagale, Nabin Pahari

**Affiliations:** 1 Department of Psychiatry, Devdaha Medical College, Bhaluhi, Rupandehi, Nepal; 2 Mental Hospital, Lagankhel, Lalitpur, Nepal; 3 Crimson College of Technology, Devinagar, Rupandehi, Nepal

**Keywords:** *health personnel*, *mental disorders*, *prevalence*, *quality of sleep*, *sleep*

## Abstract

**Introduction:**

Health personnel work under highly stressful conditions with long work hours, frequent night work, and shift duties resulting in sleep problems. Sleep problems lead to a decline in performance, cognition, memory, decision-making, medical errors and mental disorders. The study aimed to find out the prevalence of poor quality of sleep among healthcare workers in a tertiary care centre.

**Methods:**

This is a descriptive cross-sectional study conducted among healthcare workers (doctors, nurses, paramedics) of a tertiary care centre after obtaining ethical approval from the Institutional Review Committee. Data was collected from 1 October to 1 December 2023. A convenience sampling method was used. The point estimate was calculated at a 95% Confidence Interval.

**Results:**

Among 127 healthcare workers, the prevalence of poor quality of sleep was seen in 61 (48.03%) (39.34-56.72, 95% Confidence Interval). A total of 31 (50.82%) were female and 30 (49.18%) were male.

**Conclusions:**

The prevalence of poor sleep quality was found to be higher than that of other studies done in similar settings. There is a need to enhance institutional support like incorporating flexible work schedules, and regular wellness programs to alleviate poor sleep quality among healthcare workers.

## INTRODUCTION

The medical profession is considered to be a highly stressful occupation with long work hours, frequent night work, and shift duties resulting in poor quality of sleep.^1^ Reports in the literature state a high prevalence of poor sleep quality among healthcare workers throughout the world.^2-4^

Studies have shown the impact of sleep problems on performance, cognition, memory, decision-making, medical errors, quality of patient care, and medical and psychiatric problems.^5-7^ Studies on poor quality of sleep among health care professionals are limited from Nepal. Considering the prevalence and consequence of the problem, it is essential to determine the quality of sleep among healthcare workers.

The study aimed to find out the prevalence of poor quality of sleep among healthcare workers in a tertiary care centre.

## METHODS

This descriptive cross-sectional study was conducted among healthcare workers (doctors, nurses, paramedics) at Devdaha Medical College, Bhaluhi, Rupandehi, Nepal. Ethical approval was obtained from the Institutional Review Committee of same institute (Reference number: 17/2023). Data was collected from 1 October to 1 December 2023 were collected. Healthcare workers who are directly involved in the treatment of patients were included in the study. Doctors with non-clinical backgrounds, administrative staff, maintenance workers, and cleaners were excluded from the study. A convenience sampling method was used. The sample size was calculated using the formula:


$$\begin{array}{ll} \text{n} & ={\text{Z}}^{2}\times \frac{\text{p}\times \text{q}}{{\text{e}}^{\text{2}}}\\ & =1.96^{2}\times \frac{0.50\times 0.50}{0.05^{2}}\\ & =385 \end{array}$$

Where,

n = minimum required sample size
Z = 1.96 at 95 % Confidence Interval (CI)
p = prevalence taken as 50% for maximum sample size
q = i-p
e = margin of error, 5%

The sample size was adjusted for a finite population as follows:


$$\begin{array}{ll} \text{n} & =\frac{\text{n}}{1+\frac{\text{n}-1}{\text{N}}}\\ \text{n} & =\frac{\text{385}}{1+\frac{\text{385}-1}{\text{127}}}\\ & =102 \end{array}$$

Where,

n' = adjusted sample size
N = finite population

The calculated minimum required sample size was 102. By adding a 15% non-response rate, the sample size was 120. However, 127 healthcare workers were included.

The questionnaire collected information on demographics, and Pittsburgh Sleep Quality Index (PSQI). The Pittsburgh Sleep Quality Index (PSQI) is used to measure the quality and patterns of sleep in adults and participants with scores ≥5 are considered poor sleepers.^8^

Data were entered and analyzed using IBM SPSS Statistics version 24.0. The point estimate was calculated at a 95% CI.

## RESULTS

Among 127, the prevalence of poor sleep quality among healthcare workers was found to be 61 (48.03%) (39.34-56.72, 95% CI). The mean age of the respondents is 31.6±9.7 years. The mean duration of falling asleep was 27.74±23.13 minutes. A total of 31 (50.82%) were female and 30 (49.18%) were male (Table 1).

**Table 1:** Sociodemographic characteristics of healthcare workers with poor sleep quality (n = 61).

Variables		n (%)
Age group (years)	20-39	S2 (BS.2S)
	40-59	8 (13.11)
	≥60	1 (1.64)
Gender	Male	30 (49.18)
	Female	31 (50.82)
Marital status	Single	31 (50.82)
	Married	30 (49.18)
Occupation	Doctors	17 (27.87)
	Nurses	19 (31.15)
	Others	25 (40.98)

A total of 48 (78.69%) did not use sleep medications in the past month whereas 14 (11%) used sleep medications at least once in the past month. Subjective sleep quality of most of the respondents 67 (52.8%) have fairly good sleep quality. About 47 (77.05%) of respondents had daytime sleep dysfunction (Table 2).

**Table 2:** Sleep characteristics among healthcare workers with poor sleep quality (n= 61).

Sleep characteristics		n (%)
	Very good	8 (13.11)
Subjective sleep Quality	Fairly good	33 (54.10)
	Fairly bad	15 (24.59)
	Very bad	5 (8.20)
	<6	15 (24.59)
Sleep duration (hrs)	6-7	30 (49.18)
	>7	16 (26.23)
Daytime dysfunction		47 (77.05)
Use of sleep medications		13 (21.31)

## DISCUSSION

In our study, the prevalence of poor quality of sleep was seen in 61 (48.03%) which was higher than a previous study done among healthcare workers in India is 39%.^2^ The cause of poor sleep quality among healthcare workers can be attributed to hectic schedules, and increased work pressure in hospitals. A similar study done on healthcare workers in India showed the prevalence of poor sleep quality of 54.4%,^3^ which is almost similar to our study. Our study findings are also consistence with some international studies.^9-10^ The variations in the prevalence of sleep disorders might be because of different tools used in classifying sleep disorders. Studies have shown health care professionals are more vulnerable to poor sleep than the general population.^11^

The mean duration of falling asleep was 27.74±23.13 minutes. Literature suggests about 7 to 8 hours of sleep is necessary for young adults.^12^

This study has several limitations. First, the information was self-reported. Therefore, recall bias could have occurred during the data collection process. This study has limited generalizability because the sample included only one hospital located in the province-5. Therefore, further studies using nationwide systematic sampling and international comparisons are highly recommended.

## CONCLUSIONS

The prevalence of poor sleep quality was found to be higher than that of other studies done in similar settings. In addition to promoting good sleep hygiene practices, instituting measures such as rotating healthcare workers between high and low-workload areas, and conducting further research on effective strategies for preventing and treating sleep disturbances, could contribute to addressing the prevalent issue of poor sleep quality among healthcare professionals.
